# ABCE1 Is a Highly Conserved RNA Silencing Suppressor

**DOI:** 10.1371/journal.pone.0116702

**Published:** 2015-02-06

**Authors:** Kairi Kärblane, Jelena Gerassimenko, Lenne Nigul, Alla Piirsoo, Agata Smialowska, Kadri Vinkel, Per Kylsten, Karl Ekwall, Peter Swoboda, Erkki Truve, Cecilia Sarmiento

**Affiliations:** 1 Department of Gene Technology, Tallinn University of Technology, Tallinn, Estonia; 2 Competence Centre for Cancer Research, Tallinn, Estonia; 3 School of Life Sciences, Södertörn University College, S-14189, Huddinge, Sweden; 4 Department of Biosciences and Nutrition, Karolinska Institute, S-14183, Huddinge, Sweden; Wuhan University, CHINA

## Abstract

ATP-binding cassette sub-family E member 1 (ABCE1) is a highly conserved protein among eukaryotes and archaea. Recent studies have identified ABCE1 as a ribosome-recycling factor important for translation termination in mammalian cells, yeast and also archaea. Here we report another conserved function of ABCE1. We have previously described AtRLI2, the homolog of ABCE1 in the plant *Arabidopsis thaliana*, as an endogenous suppressor of RNA silencing. In this study we show that this function is conserved: human ABCE1 is able to suppress RNA silencing in *Nicotiana benthamiana* plants, in mammalian HEK293 cells and in the worm *Caenorhabditis elegans*. Using co-immunoprecipitation and mass spectrometry, we found a number of potential ABCE1-interacting proteins that might support its function as an endogenous suppressor of RNA interference. The interactor candidates are associated with epigenetic regulation, transcription, RNA processing and mRNA surveillance. In addition, one of the identified proteins is translin, which together with its binding partner TRAX supports RNA interference.

## Introduction

RNA silencing is a conserved sequence-specific mechanism regulating the gene expression in eukaryotes. This pathway functions as an innate immune response aimed at combating invading nucleic acids. In addition, RNA silencing is also a complex gene regulation pathway that controls cell differentiation and developmental processes by acting both at the transcriptional and post-transcriptional level [1,2].

Plant viruses and also some animal viruses encode suppressor proteins that are able to inhibit RNA silencing in host cells [3]. Most of the viral suppressors identified to date are highly diverse multifunctional proteins that are able to target one or more key steps of the RNA interference (RNAi) pathway [4].

In addition to viral suppressors, genomes themselves encode endogenous silencing suppressors. Very little is known about these proteins and so far endogenous regulators have been identified only in plants, *Caenorhabditis elegans* and *Dictyostelium* [5–8]. One of these proteins is RNase L inhibitor of *Arabidopsis thaliana* (AtRLI2). AtRLI2 suppresses silencing in GFP-transgenic *Nicotiana benthamiana* plants at the local as well as at the systemic level. It does not bind siRNAs and the way it suppresses RNA silencing remains unknown [9].

AtRLI2 is the plant ortholog of human ABCE1. ABCE1—also known as RNase L inhibitor (Rli1 in yeast), Pixie in *Drosophila melanogaster* and host protein 68 kDa (HP68)—belongs to the ABCE subfamily of ABC proteins that contain two nucleotide-binding domains and two N-terminal iron-sulfur clusters. Unlike most ABC domain proteins, members of this subfamily do not contain the membrane-spanning domains and are therefore not likely to be transporter proteins [10]. ABCE1 was initially identified as a negative regulator of the interferon-induced 2–5A antiviral pathway, where it functions by blocking RNase L, an enzyme responsible for the degradation of mRNA and single-stranded RNA in virus infected cells [11,12]. ABCE1 is highly conserved in archaea and eukaryotes [10,13] and has been described as essential for the viability of several organisms [14–16]. By contrast, RNase L is found only in vertebrates and therefore the question of the ABCE1 role in the rest of eukaryotes remained unanswered for almost a decade.

Recent years have brought many breakthroughs in discovering the core functions of ABCE1. This conserved protein is involved in the regulation of translation and in ribosome biogenesis through interacting with different translation initiation factors, release factors and also with ribosomal subunits in yeast, *Drosophila* and mammalian cells [17–22]. Although ABCE1 seems to be important for translation initiation, it is not well understood if its role at this stage is merely a consequence of its need for ribosomal recycling. Moreover, ABCE1 splits ribosomes not only when translation terminates but also during ribosome biogenesis and in mRNA surveillance pathways on stalled ribosomes [22–26]. Interestingly, ABCE1 is able to shuttle between nucleus and cytoplasm and is essential for nuclear export of 60S and 40S subunits in yeast [17–19].

The vast majority of recent research has focused on the central function of ABCE1 in translation and no discoveries have been made concerning the ABCE1 role in RNA silencing. As ABCE1 is a very well conserved protein and we have shown that its plant homolog, AtRLI2, acts as an endogenous suppressor of RNA silencing, we were tempted to test the role of human ABCE1 as RNA silencing suppressor.

In the current study we demonstrate that human ABCE1 is able to suppress RNA silencing in *N*. *benthamiana* plants, mammalian HEK293 cells and in the worm *C*. *elegans*. Furthermore, we identify several potential interactors which might support ABCE1 functioning as an endogenous suppressor of RNA silencing, among them translin, a protein involved in the activation of the RNA-induced silencing complex (RISC) [27].

## Materials and Methods

### Expression constructs

Binary vectors used for agroinfiltration assays were constructed as follows: pBin61 [28] was linearized with restriction enzyme *Sma*I and subsequently dephosphorylated. *AtRLI2* cDNA was cut out from the ABRC clone 232A23T7 (GeneBank Accession No. N65784) with restriction enzymes *Sma*I and *Eco105*I. Human *ABCE1* coding region was cut out from pcDNA3/RLIΔ3 (kindly provided by C. Bisbal) with restriction enzymes *Pst*I and *Not*I. The protruding ends were subsequently filled with Klenow enzyme. Ligation reaction was then performed with dephosphorylated vector and blunt ended inserts to attain pBin61-AtRLI2 and pBin61-ABCE1, respectively. pBin61 vector harboring the *GFP* gene (named here pBin-GFP) was kindly provided by D. Baulcombe and pBin61 comprising 2/3 of GFP sequence from 5’ end as inverted repeat (IR) was kindly provided by J. Burgyan and named here pBin-GFFG.

The coding regions of *ABCE1* and *Tomato bushy stunt virus* (TBSV) *P19* were PCR amplified using respectively pBin61-ABCE1 and pBin61-P19 as templates and cloned into pcDNA3.1/V5-His mammalian expression vector according to the pcDNA 3.1 Directional TOPO Expression Kit (Invitrogen) protocol. pBin61-P19 stands here for pBin61 coding for P19, a construct kindly provided by D. Baulcombe. The primers used for the generation of expression constructs were as follows: 5`-CACCATGGCAGACAAGTTAA–3`and 5`-ATCATCCAAGAAAAAGTAGTTTCC–3`for ABCE1, 5`-CACCATGGAACGAGCTATAC–3`and 5`-CTCGCTTTCTTTTTCGAAGGT–3`for P19. The resulting plasmids pABCE1-V5 and pP19-V5 contain C-terminal V5 and His tags. The expression constructs were verified by sequencing and *in vitro* transcription-translation assay (Promega).

pULK3FLAG and siRNA1pSUPER constructs—here renamed as siRNA(ULK3)—are described in [29] and [30], respectively. Construct siRNA(Fu)pSUPER—here renamed as siRNA(X)—is described in [31]. Empty vectors pSUPER (OligoEngine) and pcDNA3.1/myc-His (Invitrogen) were used as controls.

To create constructs pAS1 and pCS1 expressing *C*. *elegans* ERI-1 and human ABCE1, respectively, under the control of the *C*. *elegans myo-3* promoter, *C*. *elegans eri-1* cDNA and human *ABCE1* cDNA were inserted into pPD96.52 [32]. pPD96.02 [32] was used to express *C*. *elegans unc-54*::*NLS*::*gfp*. To generate the GFP-specific construct for RNAi by feeding, *GFP* coding sequence was inserted into the L4440 backbone [32].

### Plant material and agroinfiltration

Wild-type *N*. *benthamiana* and *N*. *benthamiana* GFP-transgenic line 16c (kind gift of D. Baulcombe) were grown in a plant chamber at 22°C or 25°C with a 16-h photoperiod. 5-week old plants were used for agroinfiltration. All binary plasmids were transformed into *Agrobacterium tumefaciens* strain C58C1 harboring pCH32 [33]. Recombinant *A*. *tumefaciens* strains were incubated and infiltrated as described in [34] adjusting the final densities to OD_600_ = 0.5 with the exception of pBin61-GFFG that had a final density of OD_600_ = 0.05. *A*. *tumefaciens* carrying the inducer (pBin61-GFP or pBin61-GFFG) was mixed in 1:1 ratio with bacterium containing pBin61-AtRLI2 or pBin61-ABCE1 or the empty vector pBin61. Six independent experiments were carried out, each including 4–7 infiltrated plants for each mixture. GFP fluorescence was monitored using long-wave ultraviolet (UV) lamp (Black-Ray B-100AP, Ultraviolet Products). Plants were photographed with a Nikon p7000 camera using a yellow UV(O) filter (Tokina) and images were processed with Adobe Photoshop CS5.

### Mammalian cell culture and transfection

HEK293 cells (ATCC Number: CRL-1573) were grown in Minimum Essential Medium (MEM) supplemented with 10% fetal bovine serum, 100 U/ml penicillin and 0.1 mg/ml streptomycin (all purchased from PAA) at 37°C and 5% CO_2_. For the *ULK3* RNAi assays, cells were co-transfected with expression constructs pULK3FLAG encoding FLAG-tagged ULK3, siRNA(ULK3) encoding *ULK3*-specific shRNA and pABCE1-V5 or pP19-V5 using polyethylenimine (PEI) (Inbio). Constructs pULK3FLAG, siRNA(ULK3), pABCE1-V5/ pP19-V5 or their respective empty vectors were used in ratio 0.05:0.5:1.5. Approximately 0.25 μg of DNA per 1 cm^2^ of plate surface area was used. DNA and PEI were diluted in 100 μl of MEM in mass ratio 1:2 and incubated for 10 min at room temperature (RT). The cells were washed with phosphate buffered saline (PBS) prior to transfection, and DNA/PEI complex was added to the cells in growth medium without supplements for 3 h. The medium was changed for normal growth medium, and the cells were propagated for an additional 30 h prior to lysis. For immunoprecipitations (IP), HEK293 cells were transfected with 20 μg of pABCE1-V5 or empty vector on 10-cm plates using PEI with DNA to reagent ratio 1:2.

### *C*. *elegans* transgenic strains and RNAi inhibition assays

*C*. *elegans* strains and genotypes (in brackets) used in this study are as follows, whereby N2 designates a wild-type background: OE4201 (N2; ofEx851 [*unc-54*::*NLS*::*gfp; rol-6(su1006)*]; AS2 (N2; asEx2 [*myo-3*::*eri-1*; *myo-2*::*dsRed*]); AS3 (N2; asEx3 [*myo-3*::*eri-1*; *myo-2*::*dsRed*]); AS4 (N2; asEx4 [*myo-3*::*ABCE1*; *unc-122*::*dsRed*]); AS5 (N2; ofEx851; asEx2); AS6 (N2; ofEx851; asEx3); AS7 (N2; ofEx851; asEx4). The *unc-54*::*NLS*::*gfp* reporter was created by injection of the following mix: 5 ng/μl pPD96.02, 20 ng/μl pRF6 [35]. For lines expressing RNAi suppressors, 10 ng/μl of *myo-3*::*eri-1* (pAS1) were co-injected with *myo-2*::*dsRed*, and 15 ng/μl of *myo-3*::*ABCE1* (pCS1) were co-injected with *unc-122*::*dsRed*. In all cases, the DNA concentration in the injection mixes was brought to 100 ng/μl using digested yeast genomic DNA.

*E*. *coli* strain HT-115, freshly transformed with GFP-L4440 or L4440 plasmids was used to seed Nematode Growth Medium (NGM) plates supplemented with tetracycline and ampicillin at standard working concentrations. Double transgenic worms were picked onto a separate NGM plate, allowed to lay eggs and progeny expressing the *rol-6(su1006)* marker at the L3 stage was placed on the *GFP* RNAi plates for 24 hours at 20°C. At this point, worms expressing both the *rol-6* and the red fluorescent markers, *i*.*e*. containing the *myo-3*::*eri-1* or the *myo-3*::*ABCE1* transgenes, were designated as “double transgenic” and imaged as the test group, whereas *rol-6*-only worms were the control group. At the same time, *rol-6*-only worms on *GFP* RNAi were used as a general control for RNAi efficacy of each batch of the RNAi plates. The L4440 plasmid was used as a control in this case.

Live worms were anesthetized with 15 mM NaN_3_ and imaged using Axioplan (Zeiss) microscope equipped with an FITC filter and an LCD camera (Hamamatsu). For each experiment all worms were imaged using identical camera and microscope settings. The GFP intensity was quantified using ImageJ [36]. The average GFP intensity in GFP-positive muscle cell nuclei was measured as the average intensity along a segmented line drawn inside each nucleus. At least 10 worms were examined per strain per condition, and at least 10 nuclei per animal. Two independent transgenic lines were tested for ERI-1 expression and one for ABCE1 expression, in at least two experiments involving independent RNAi plate batches. The statistical analysis of the GFP intensity data was performed using R [37].

### RNA extraction and northern blot analysis

Total RNA was extracted from infiltrated leaf patches as described previously [38] and 10 μg was used for GFP mRNA northern blot analysis as reported in [9]. For the detection of GFP siRNAs, RNA extraction was performed with TRIzol Reagent (Invitrogen) following manufacturer’s instructions. 30 μg of total RNA was analyzed according to [39]. Radioactive signals were scanned and analyzed by Personal Molecular Imager FX (BioRad) after 30 min for mRNA and 24 h for siRNA.

### Western blot analysis

Mammalian cells were lysed in RIPA buffer (50 mM Tris–HCl [pH 7.4], 150 mM NaCl, 2 mM EDTA, 1% NP-40, 0.1% sodium dodecyl sulfate) containing ProteoBlock Protease Inhibitor Cocktail (Thermo Scientific) and incubated on ice for 10 min. Cell lysates were centrifuged for 3 min at 4°C and 15,000 g. Supernatants were mixed with Laemmli Sample Buffer and denaturated for 5 min at 96°C. Proteins were separated on 10% polyacrylamide gel and transferred to polyvinylidene difluoride membrane (Millipore). The membrane was blocked with 5% non-fat dry milk (AppliChem) in PBS containing 0.1% Tween 20 (Sigma-Aldrich). The antibodies were diluted in PBS containing 0.1% Tween 20 and 1% non-fat dry milk as follows: mouse monoclonal anti-V5 (1:2000, Invitrogen, catalog #R960–25), mouse monoclonal anti-FLAG M2-Peroxidase (1:5000, Sigma-Aldrich, catalog #A8592), rabbit polyclonal anti-actin (1:1000, Santa Cruz Biotechnology, catalog #sc-7210), HRP-conjugated goat anti-mouse IgG (1:3000, Thermo Scientific, product #32430) and HRP-conjugated goat anti-rabbit IgG (1:3000, Thermo Scientific, product #32460). The proteins were visualized using SuperSignal West Femto Chemiluminescense Substrate kit (Thermo Scientific).

ULK3FLAG western blot images were quantified with ImageJ software [36] and the data was expressed as mean of three independent experiments ± standard deviations. Statistical analysis was carried out using Microsoft Excel and JMP 10.0 software. Data was analyzed by mean centering and autoscaling as suggested in [40]. Two-tailed p-values were calculated using ANOVA with Dunnett’s post-hoc comparison.

### Co-immunoprecipitation

HEK293 cells were collected 28 h post-transfection and lysed with buffer (25 mM Tris-HCl [pH 7.4], 150 mM NaCl, 1 mM EDTA, 1% Triton X-100, 5% glycerol, protease inhibitors cocktail Complete (Roche)). Lysates were centrifuged for 25 min at 4°C and 13,400 g, and the supernatants were used for IP. Supernatants were incubated with 2 μg mouse monoclonal anti-V5 antibody (Invitrogen, catalog #R960–25) for 1 h at 4°C with gentle agitation. The protein-antibody complexes were incubated with ethanolamine-blocked protein G sepharose beads (GE Healthcare) overnight at 4°C with gentle agitation. After IP the beads were washed three times with ice cold PBS and the precipitated immune complexes were analyzed by mass spectrometry.

### Mass spectrometry

For mass spectrometry analysis, three different sample preparation methods were used—in-gel digestion (experiment was carried out twice), in-solution digestion and Filter Aided Sample Preparation (FASP). For in-gel digestion, precipitated proteins were eluted with LDS sample buffer (Invitrogen) and separated by SDS-PAGE using a 4–12% NuPAGE Bis-Tris gel system (Invitrogen). The gel was stained with SimplyBlue SafeStain (Invitrogen) for 1 h at RT and washed in distilled H_2_O prior to excision of equal slices. Gel pieces were destained in 100 mM ammonium bicarbonate/acetonitrile (1:1, v/v) and dehydrated with 100% acetonitrile. Proteins were reduced and alkylated, first by the addition of 10 mM dithiothreitol (DTT), and then by the addition of 50 mM iodoacetamide. After alkylation, proteins were digested with 2.5 ng/μl trypsin overnight at 37°C. Peptides were extracted from gel pieces using 5% formic acid/acetonitrile (1:2, v/v) and sample volume was reduced in SpeedVac to about 25% of the starting volume. 1/10 of 5% acetic acid was added to the sample and peptides were purified on StageTip columns as described in [41]. For in-solution digestion, immunocomplexes were eluted with 1% SDS and precipitated with methanol and chloroform. Precipitated proteins were resuspended in 7 M urea/2 M thiourea solution and reduction/alkylation step was performed using DTT and iodoacetamide. Proteins were first digested with Lys-C for 3 h at RT and then with trypsin overnight at RT. 1/10 of 10% trifluoroacetic acid was added to the sample and peptides were purified on StageTip columns. For FASP, precipitated proteins were eluted with 1% SDS, out-dilution of SDS with urea and protein digestions were performed with 10 k filter as described in [42]. Peptides were again purified on StageTip columns.

Purified peptides were resuspended in 0.5% acetic acid and liquid chromatography-tandem mass spectrometry (LC-MS/MS) analysis of two technical replicates was performed using an Agilent 1200 series nanoflow system (Agilent Technologies) connected to a LTQ Orbitrap mass-spectrometer (Thermo Scientific) equipped with a nanoelectronspray ion source (Proxeon) as described in [43].

Raw data files were analyzed with the MaxQuant software package (version 1.2.7.4) [44]. Generated peak lists were searched using the Andromeda search engine (built into MaxQuant) against the UniProt human database. MaxQuant search was performed with full tryptic specificity, a maximum of two missed cleavages and a mass tolerance of 0.5 Da for fragment ions. Carbamidomethylation of cysteine was set as a fixed modification and methionine oxidation and protein N-terminal acetylation were set as variable modification. The required false discovery rate was set to 1% both for peptide and protein levels and the minimum required peptide length was set to six amino acids. Candidate interacting proteins were those present in at least two experimental samples with experimental sample and control sample intensity ratio cut-off set at > 2. Proteins that could be linked to transcriptional (TGS) or post-transcriptional silencing (PTGS) and that have putative homologs in *A*. *thaliana* and in *C*. *elegans* (according to [45]) were chosen as ABCE1 potential binding partners.

## Results

### ABCE1 suppresses RNAi of *GFP* in *Nicotiana benthamiana* plants

**Fig 1:**
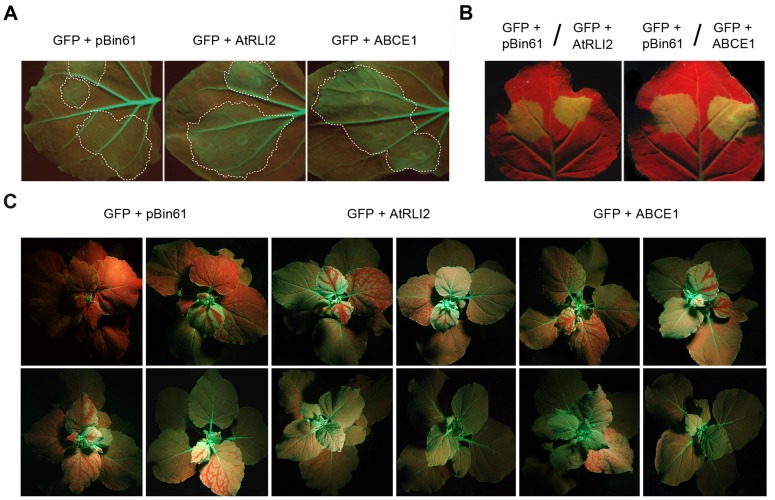
ABCE1 suppresses RNAi of *GFP* in *N*. *benthamiana*. 5-week old GFP-transgenic *N*. *benthamiana* 16c and wild-type *N*. *benthamiana* plants were co-infiltrated with *A*. *tumefaciens* harboring the RNA silencing inducer (pBin61-GFP) and pBin61-AtRLI2 or pBin61-ABCE1 or pBin61 as control. **(A)** Representative infiltrated GFP-transgenic *N*. *benthamiana* 16c leaves were photographed under UV light at 5 dpi. Strong GFP fluorescence in the patches infiltrated with pBin61-GFP/pBin61-AtRLI2 (indicated as “GFP + AtRLI2”) and pBin61-GFP/pBin61-ABCE1 (indicated as “GFP + ABCE1”) mixtures revealed the suppression of local GFP silencing. Control leaf infiltrated with pBin-GFP/pBin61 mixture (indicated as “GFP + pBin61”) displayed weak GFP fluorescence. Infiltrated patches are circled in white. **(B)** Representative infiltrated wild-type *N*. *benthamiana* leaves were photographed under UV light at 5 dpi. Strong GFP fluorescence in the patches infiltrated with pBin61-GFP/pBin61-ABCE1 or pBin61-GFP/pBin61-AtRLI2 indicates the suppression of local GFP silencing. **(C)** *N*. *benthamiana* 16c plants showing systemic GFP silencing in the uppermost leaves. Representative plants were photographed under UV light at 21 dpi. Plants infiltrated with pBin61-AtRLI2 or pBin61-ABCE1 displayed less silenced tissue compared to the control (pBin61).

To investigate whether human ABCE1 is able to suppress RNA silencing in plants, we used a transient expression system in *N*. *benthamiana* harboring a stably expressing GFP transgene (16c line). Leaves were co-infiltrated with *A*. *tumefaciens* carrying pBin61-GFP (*GFP* gene as silencing inducer) and pBin61-ABCE1. As controls, we co-infiltrated *A*. *tumefaciens* containing pBin61-GFP and pBin61-AtRLI2 or the empty vector pBin61. The suppression activity was assessed according to [9]. At 5 dpi the patches infiltrated with pBin61-GFP/pBin61 mixture displayed a weak GFP fluorescence due to RNA silencing. In contrast, tissues infiltrated with pBin61-GFP/pBin61-ABCE1 mixture showed high intensity of GFP fluorescence, similarly to pBin61-GFP/pBin61-AtRLI2, indicating that ABCE1, as well as AtRLI2, suppresses local *GFP* RNAi (Fig. 1A). Using an *in vivo* imaging system we quantified the GFP fluorescence in the infiltrated patches and verified that the presence of either ABCE1 or AtRLI2 enhanced the expression of GFP (S1 Fig.). Thereafter, we also examined ABCE1 as a suppressor in wild type *N*. *benthamiana* plants, considered a weak silencing system, since RNA silencing in this case targets only ectopically expressed GFP. We found that the level of GFP expression was likewise enhanced in the presence of ABCE1, as well as in the presence of AtRLI2, compared to the empty plasmid control (Fig. 1B).

Further, we explored if ABCE1 is able to suppress systemic RNA silencing. For this reason, we infiltrated 16c *N*. *benthamiana* plants as described above and followed the spread of silencing for three weeks. Systemic silencing of GFP can be clearly observed under UV light as emerging red tissue in the leaves above the infiltrated ones. At 21 dpi, plants co-infiltrated with pBin61-GFP and pBin61-ABCE1 displayed significantly less silenced tissue compared to empty plasmid pBin61 (Fig. 1C). Only 41% and 31% of the plants infiltrated with pBin61-GFP/pBin61-ABCE1 and pBin61-GFP/pBin61-AtRLI2 mixtures, respectively, were silenced at the uppermost leaf compared to 78% in the case of pBin61-GFP/pBin61. Thus, ABCE1 suppressed *GFP* RNAi at the systemic level.

**Fig 2:**
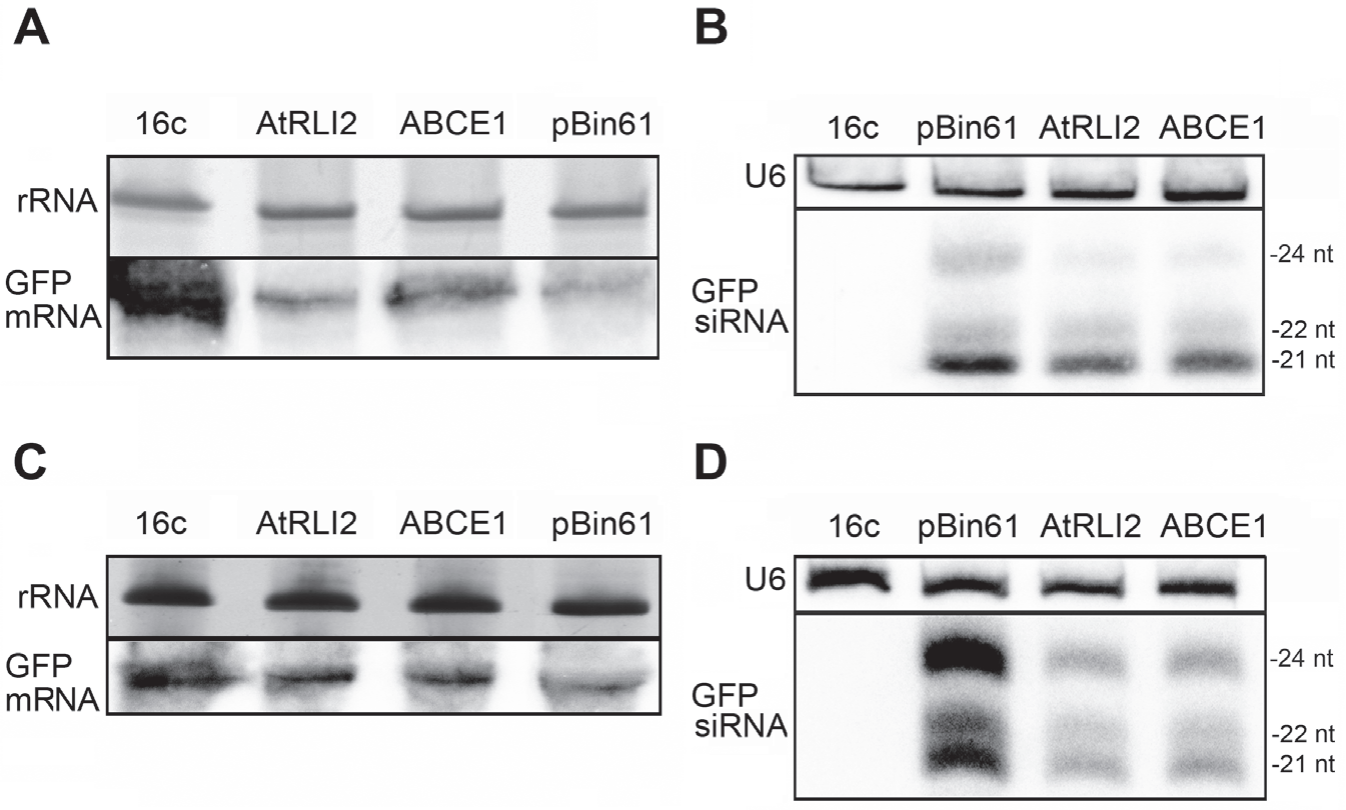
Northern blot analysis showing the suppression of *GFP* RNA silencing in *N*. *benthamiana* by ABCE1. **(A)** GFP-transgenic *N*. *benthamiana* 16c leaves were infiltrated with *A*. *tumefaciens* harboring pBin61-GFP together with *A*. *tumefaciens* carrying pBin61-AtRLI2 or pBin61-ABCE1 or empty vector (pBin61), as indicated on the upper part of the panel. Total RNA was extracted from the infiltrated patches and analyzed by northern blot. Levels of GFP mRNA in the patches infiltrated with pBin61-GFP/pBin61-AtRLI2 or pBin61-GFP/pBin61-ABCE1 were higher than in the case of pBin61. 16c indicates non-infiltrated leaf. GFP mRNAs were detected using [α-^32^P] UTP-labeled antisense GFP transcripts. Ethidium bromide staining of rRNA was used as loading control. **(B)** Total RNA was extracted from the infiltrated patches and analyzed by northern blot as indicated before. GFP siRNA levels were reduced in the presence of AtRLI2 or ABCE1 compared to the control (pBin61). 16c indicates non-infiltrated leaf. For the detection of GFP siRNAs [γ-^32^P] ATP end-labeled GF-probe was used. U6 stands for U6 snRNA used as loading control. **(C)** pBin61-GFFG was infiltrated as RNA silencing inducer instead of pBin61-GFP. The northern blot analysis of GFP mRNAs was performed as in (A) and shows higher levels in the presence of AtRLI2 or ABCE1 than in the case of the empty vector. **(D)** The accumulation of GFP siRNAs in the patches infiltrated with pBin61-GFFG/pBin61-AtRLI2, pBin61-GFFG/pBin61-ABCE1 or pBin61-GFFG/pBin61 was analyzed as in (B). GFP siRNA levels were reduced in the presence of AtRLI2 or ABCE1 as compared to the control (pBin61).

To confirm the suppression activity of ABCE1, we analyzed by northern blot GFP mRNA levels and the accumulation of GFP-specific siRNAs—indicators of RNA silencing—in the infiltrated patches. We found that GFP mRNA levels were increased in the patches infiltrated with either pBin61-GFP/pBin61-ABCE1 or pBin61-GFP/pBin61-AtRLI2 mixtures with respect to pBin61-GFP/pBin61 (Fig. 2A). Accordingly, accumulation of GFP siRNAs was high in the case of empty plasmid, but was significantly reduced, particularly 24 nt long siRNAs, in the presence of ABCE1 or AtRLI2 (Fig. 2B). In addition, we challenged the suppressor efficiency of ABCE1 using conditions that favor strong GFP silencing: higher temperature and an IR construct as inducer [46,47]. A pBin61-GFFG construct comprising partial sequence of GFP, was agroinfiltrated together with pBin61-ABCE1 or pBin61-AtRLI2 into 16c *N*. *benthamiana* plants kept at 25°C. RNA analysis demonstrated that ABCE1 reduced the degradation of GFP mRNA and strongly affected the accumulation of GFP siRNAs in this system (Fig. 2C and 2D). Overall, these experiments show that ABCE1 suppresses local and systemic RNA silencing in plants by reducing siRNA accumulation.

### ABCE1 suppresses RNAi mediated silencing of *ULK3* in mammalian HEK293 cells

**Fig 3:**
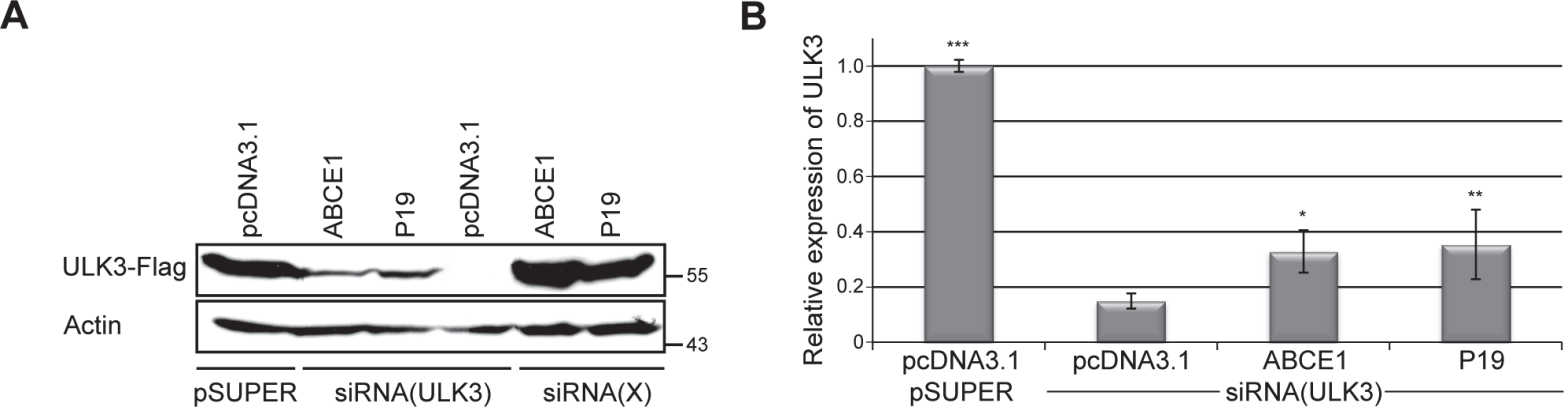
ABCE1 suppresses RNAi mediated silencing of *ULK3* in HEK293 cells. FLAG-tagged ULK3 was expressed in HEK293 cells in combination with empty vectors pcDNA3.1 and pSUPER or with siRNA(ULK3) or scrambled siRNA(X) and plasmids encoding either ABCE1 or P19 proteins. Cells were analyzed 30 h post-transfection. **(A)** FLAG-tagged ULK3 and actin (loading control) were detected by western blotting. The blot shown is representative for three independent experiments. ABCE1 and P19 were able to increase ULK3 expression levels in silenced cells. ABCE1 and P19 did not have any significant effect on ULK3 expression level in siRNA(X) transfected (non-silenced) cells. Molecular masses (in kDa) are shown on the right. **(B)** Quantification of relative ULK3 expression levels derived from three independent experiments. ULK3 expression levels were normalized to actin expression levels. The means relative to the levels of non-silenced ULK3 (cells transfected with empty vectors) are shown. Error bars indicate standard deviations. ABCE1 and P19 rescued ULK3 expression level significantly (*p = 0.0835, **p = 0.0461).

To examine whether ABCE1 is able to suppress RNA silencing in mammalian cells, we overexpressed ABCE1 together with ULK3 (Unc-51-like serine/threonine kinase controlling Gli proteins in the Sonic hedgehog pathway [29]) and suppressed ULK3 expression by RNAi. For this, we co-transfected pABCE1-V5, pULK3FLAG and siRNA(ULK3) constructs in HEK293 cells and analyzed ULK3 protein levels with western blot 30 h post-transfection. TBSV P19, which has been shown to function effectively in mammalian cells [48,49], was used as the positive control for RNAi suppression. Expression of V5-tagged ABCE1 and P19 was detected with anti-V5 antibody (S2 Fig.) while expression of FLAG-tagged ULK3 was detected using anti-FLAG antibody. The experiment was repeated three times, and the results of a representative experiment are shown in Fig. 3A. ULK3 overexpressed protein levels in the three independent experiments were quantified with ImageJ software (Fig. 3B). Cells expressing ABCE1-V5 showed higher ULK3 transient expression level than mock-transfected cells (Fig. 3A and B). The effect of ABCE1 on *ULK3* RNAi was comparable to the effect of P19 (Fig. 3A and B). While the expression of both proteins resulted only in a minor increase of ULK3 expression levels, the results were reproducible and statistically significant (Fig. 3B).

In order to exclude the possibility that ABCE1 induces ULK3 expression levels despite silencing, we transfected the examined protein expression constructs or empty vector together with pULK3FLAG and siRNA(X), a plasmid generating scrambled siRNAs. No significant changes in ULK3 expression levels were observed (S3A Fig.). Furthermore, we included an additional reporter, Firefly luciferase, to our test system. Cells were transfected with pABCE1-V5, pP19-V5 or pcDNA3.1 together with pULK3FLAG, siRNA(X) and pFLuc, a plasmid encoding Firefly luciferase. Neither ULK3 expression analysis nor luminescence measurements showed any significant changes in reporter gene translation (S3B and C Fig.). We therefore conclude that ABCE1 suppresses RNAi of *ULK3* in HEK293 cells.

### ABCE1 suppresses RNAi of *GFP* in the worm *C*. *elegans*

To address whether human ABCE1 can function as a *bona fide* RNAi inhibitor in a heterologous animal context, we tested if it can inhibit RNAi in the worm *C*. *elegans*, as this model organism has a robust and well characterized RNAi pathway and has frequently been used for studies of RNAi [50,51].

**Fig 4:**
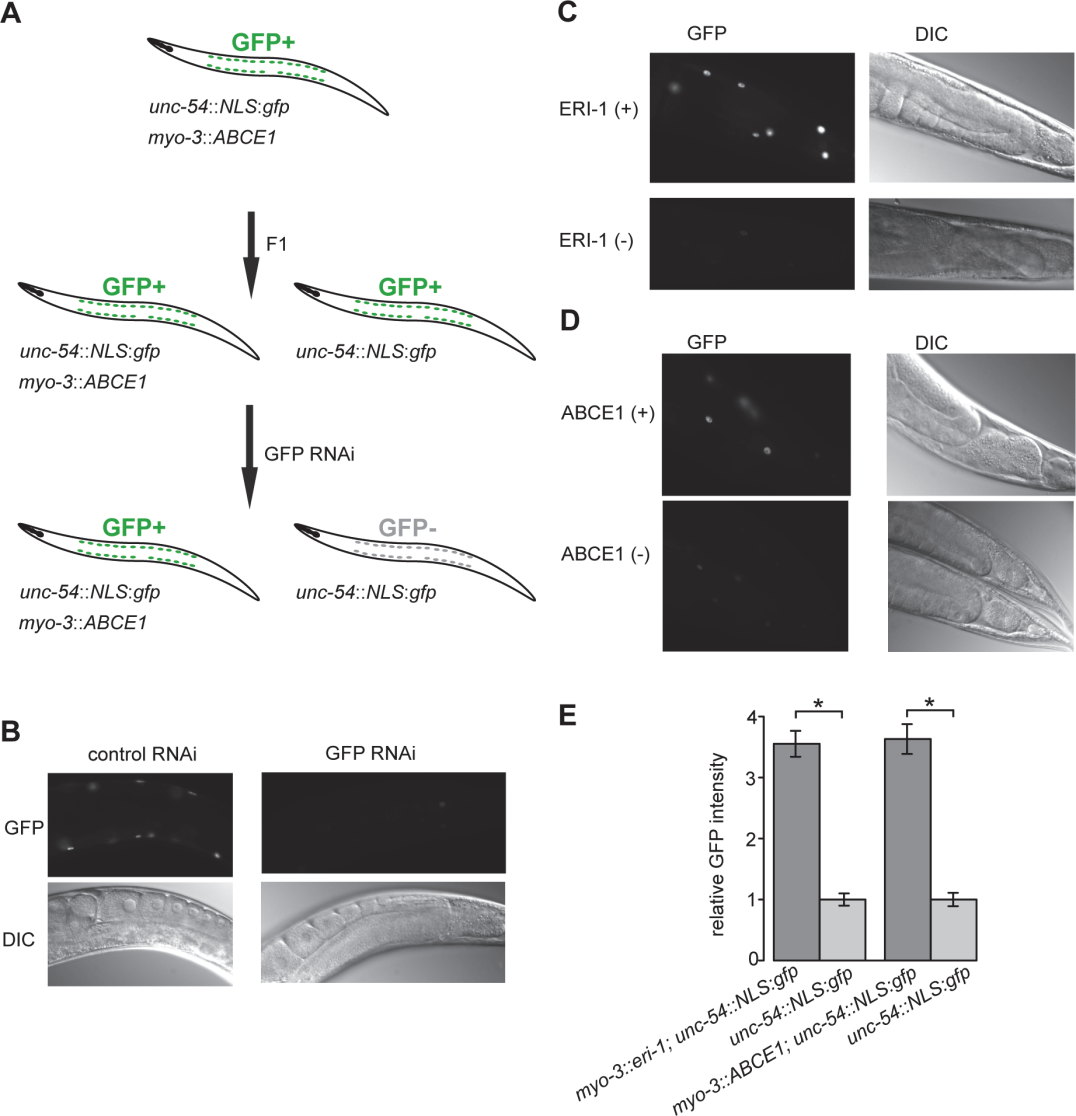
ABCE1 suppresses RNAi of *GFP* in *C*. *elegans*. **(A)** Schematic representation of the *in vivo* RNAi inhibition assay—the transgenes expressed and the GFP status are indicated. (**B)** Representative photomicrographs of the posterior part of animals expressing the NLS::GFP reporter in body wall muscles, treated with *GFP*-specific RNAi for 24 h at 20°C; control RNAi—empty vector. (**C)** Representative photomicrographs of the posterior part of animals expressing *C*. *elegans* ERI-1 and the NLS::GFP reporter in body wall muscles, treated with *GFP*-specific RNAi for 24 h at 20°C; ERI-1(+)—animals carrying the *myo-3*::*eri-1* transgene; ERI-1(-)—animals not carrying the *myo-3*::*eri-1* transgene. (**D)** Representative photomicrographs of the posterior part of animals expressing ABCE1 and the NLS::GFP reporter in body wall muscles, treated with *GFP*-specific RNAi for 24 h at 20°C. ABCE1(+)—animals carrying the *myo-3*::*ABCE1* transgene; ABCE1(-)—animals not carrying the *myo-3*::*ABCE1* transgene. (**E)** Relative GFP fluorescence intensity of the NLS::GFP reporter in worms expressing ERI-1 and ABCE1 and treated with *GFP*-specific RNAi for 24 h at 20°C. The ratio of the GFP signal intensity in worms expressing ERI-1 or ABCE1 and the NLS::GFP reporter compared to reporter alone is presented. Results from a representative experiment are shown (n > 100). Error bars represent the 95% confidence interval for the mean. Asterisks denote a statistically significant increase of the GFP signal intensity in worms expressing ERI-1 or ABCE1 and the NLS::GFP reporter as compared to the reporter alone (p < 0.0001 by two-tailed Student’s *t*-test), showing that ERI-1 and ABCE1 are able to suppress *GFP* RNAi.

We developed an *in vivo* RNAi inhibition assay, whereby GFP fused to the nuclear localization signal (NLS) from SV40, expressed under the control of a body wall muscle specific promoter *unc-54* (*unc-54*::*NLS*::*gfp*), was used as a reporter (Fig. 4A). 24 h after the worms had been subjected to GFP-specific RNAi by feeding, the GFP expression was effectively silenced (Fig. 4B). To test the functionality of the assay, we asked whether the known *C*. *elegans* endogenous RNAi inhibitor ERI-1 can suppress the *GFP*-specific RNAi [52]. We constructed a double transgenic strain, expressing ERI-1 under the control of a body wall muscle specific *myo-3* promoter, and the *unc-54*::*NLS*::*gfp* reporter on separate extrachromosomal arrays. Upon expression of ERI-1, the GFP signal in worms subjected to *GFP*-specific RNAi was significantly stronger than in worms not expressing ERI-1 (Fig. 4C and E). Similarly, expression of human ABCE1 in *C*. *elegans* body wall muscles lead to increased GFP levels of the *unc-54*::*NLS*::*gfp* reporter in worms subject to *GFP*-specific RNAi, compared to worms not expressing human ABCE1 (Fig. 4D and E). In both cases, the expression level of the *unc-54*::*NLS*::*gfp* reporter in worms not subject to *GFP*-specific RNAi was the same irrespective of the presence of the extrachromosomal array carrying either the ERI-1 or the ABCE1 expressing construct (data not shown). Taken together, our results indicate that both *C*. *elegans* ERI-1 and human ABCE1 inhibited *GFP*-specific RNAi similarly, when expressed in body wall muscle cells in *C*. *elegans*.

### Identification of potential ABCE1-interacting proteins

**Fig 5:**
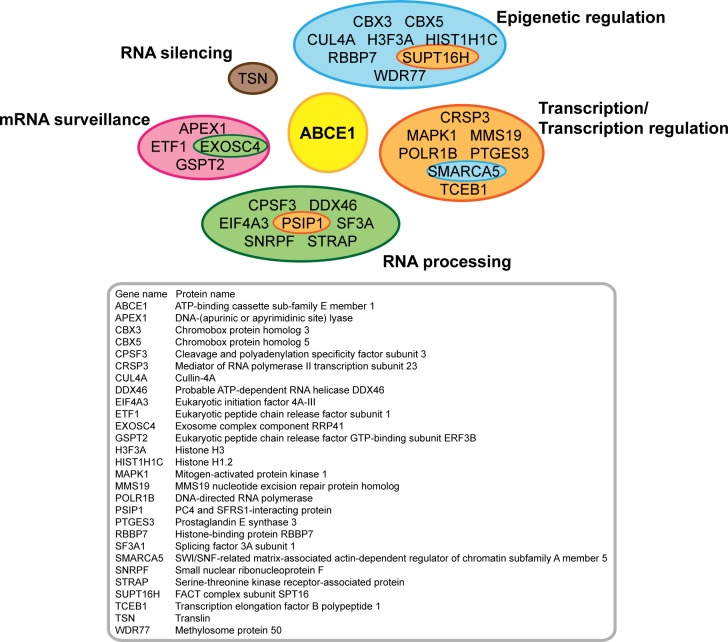
Candidate ABCE1-interacting proteins. HEK293 cells expressing V5-tagged ABCE1 and mock-transfected cells were subjected to IP with anti-V5 antibody. The protein content of the obtained immune complexes was analyzed using LC-MS/MS. Potential ABCE1 binding partners (proteins present in at least two experimental samples with experimental and control sample intensity ratio > 2, cf. S1 Table) that could be linked to RNA silencing and have putative homologs in *A*. *thaliana* and *C*. *elegans* (cf. S2 Table) were grouped according to their biological functions: epigenetic regulation (blue), transcription/transcription regulation (orange), RNA processing (green), mRNA surveillance (pink) and RNA silencing (brown). Four proteins were categorized into two groups and are therefore marked differently: EXOSC4—epigenetic regulation and transcription/transcription regulation, PSIP1—RNA processing and transcription/transcription regulation, SMARCA5—transcription/transcription regulation and epigenetic regulation, SUPT16H—epigenetic regulation and transcription/transcription regulation. ABCE1 is depicted in yellow.

To date, it is known that human ABCE1 or its orthologs interact with different ribosomal proteins, translation initiation and termination factors [17–21,23]. In order to identify novel ABCE1-associated proteins, V5-tagged ABCE1 was overexpressed in HEK293 cells and immunoprecipitated using anti-V5 antibody. As a negative control we immunoprecipitated pcDNA3.1 transfected cells with anti-V5 antibody. The protein content of the obtained immune complexes was analyzed using LC-MS/MS. The candidate ABCE1-interacting proteins present in at least two experimental samples are presented in S1 Table. Mass-spectrometry analysis identified a number of proteins (with putative homologs in *A*. *thaliana* and *C*. *elegans*) that could be linked to TGS or PTGS and might support ABCE1 functioning as an endogenous suppressor of RNAi (S2 Table). These potential binding partners were grouped according to their biological functions: epigenetic regulation, transcription/transcription regulation, RNA processing, mRNA surveillance and RNA silencing (Fig. 5). One of the identified proteins was translin, which together with its binding partner TRAX forms the C3PO (component 3 promoter of RISC) complex that is known to activate RISC [27].

## Discussion

ABCE1 is a very well conserved protein in eukaryotes and archaea and is considered to be a multifunctional protein essential for the viability of several organisms [14–16]. Initially described as a negative regulator of the 2–5A antiviral pathway, human ABCE1 is at present known to play an important role in several steps of translation, in ribosome biogenesis and recycling. It is assumed that these functions are conserved among eukaryotes and archaea, as they have also been described for some ABCE1 orthologs [13,53]. However, up to now there is no evidence that an ABCE1 plant ortholog is involved in translation. We propose here that another possible common role of ABCE1 across kingdoms is linked to RNA silencing, since we have reported that the ABCE1 plant ortholog AtRLI2 is an endogenous suppressor of RNAi [9].

To evaluate the hypothesis that functioning as an endogenous suppressor is conserved for ABCE1, we analyzed human ABCE1 effect on RNA silencing in *N*. *benthamiana* plants. Firstly, we observed that ABCE1 is able to suppress GFP-induced silencing in *N*. *benthamiana* at the local and at the systemic level. Furthermore, we found that the effect of human ABCE1 is comparable to the effect of AtRLI2, its plant ortholog. RNA analysis showed that the expression of ABCE1 leads to the accumulation of GFP mRNA and reduction of GFP siRNAs. Interestingly, the reduction in 24 nt siRNA levels was most significant. When using a stronger silencing system (IR construct as inducer and higher temperature), the siRNA levels decreased similarly in the presence of ABCE1. Again we observed that the levels of 24 nt siRNAs were most affected. 24 nt siRNAs are associated with systemic spread of silencing and are candidates for the long-range phloem entry signal because viral proteins that block systemic silencing also prevent accumulation of the 24 nt siRNAs [34,54]. We indeed observed a strong effect of ABCE1 at the systemic silencing level. Moreover, this class of siRNAs is the only mobile small RNA species that is active in RNA-dependent DNA methylation (RdDM) and TGS [55].

To test whether the ABCE1 ability to suppress RNA silencing is not only plant specific, we analyzed the effect of ABCE1 on exogenous ULK3 silencing in mammalian HEK293 cells. ABCE1 expression resulted in a reproducible and statistically significant upregulation of ULK3 protein level. Moreover, we compared ABCE1 with tombusvirus P19, a well-characterized viral RNA silencing suppressor which has been shown to function effectively in HeLa and HepG2 cells [48,49], and found the ABCE1 effect to be only slightly weaker.

After showing that ABCE1 is able to function as an endogenous RNA silencing suppressor in plants and mammalian cells, whereby the effectiveness seems to differ in the tested systems, we analyzed ABCE1 suppressor function in the worm *C*. *elegans*. We found that the expression of ABCE1 leads to increased GFP levels in worms subjected to *GFP*-specific RNAi. Additionally, we observed that ABCE1 ability to rescue GFP levels is comparable to ERI-1, a well-described *C*. *elegans* endogenous RNA silencing suppressor that forms a complex with Dicer [8].

It is noteworthy that in *C*. *elegans* ABCE1 seems to act as a strong silencing suppressor and the same appears to be the case for *N*. *benthamiana*, at least at the systemic level. This might indicate that ABCE1 predominantly affects a step in the RNAi pathway that is similar in plants and *C*. *elegans*, for instance the ability to amplify siRNAs. In nematodes and plants, but not in mammals, RNA-dependent RNA polymerases (RdRPs) are required for RNA silencing pathways acting in the cytoplasm and at the chromatin level. As a result of the RdRP-mediated mechanisms, from a single aberrant RNA species (only in the case of plants) or primary siRNA molecules, many dsRNAs/secondary siRNAs can be generated and these are then able to silence even more target molecules. In addition, in plants and *C*. *elegans* the silencing signal is able to spread systematically through the organism [54]. Thus, we speculate that ABCE1 is involved to some extent in siRNA amplification and/or systemic movement of the silencing signal.

We next aimed to understand the mechanisms underlying ABCE1 role in RNA silencing pathway. In the case of AtRLI2, we have previously shown that it does not bind siRNAs [9]. Hence, the mechanism how ABCE1 and its orthologs work as negative regulators of RNAi might be through interaction with other proteins that function in different steps of RNAi pathways. To address this speculation, we co-immunoprecipitated V5-tagged ABCE1 in mammalian HEK293 cells and analyzed the immune complexes with mass spectrometry. From the obtained list of proteins we selected the ones that could be linked to RNA silencing and have putative homologs in *A*. *thaliana* and *C*. *elegans*. We then grouped these potential binding partners according to their biological functions. Keeping in mind that different RNAi pathways are interconnected by competing for substrates, effector proteins and by cross-regulating each other, we firstly chose proteins associated with transcription and epigenetic regulation. These putative binding partners might be linked to TGS as in different organisms TGS is associated with RdDM, nucleosomal histone tail modifications, heterochromatin formation and transcription inhibition [56]. Recent years have brought growing evidence suggesting that different RNAi pathways interact with RNA surveillance and processing [57,58]. Due to these possible connections between different RNA-associated pathways, we secondly selected proteins related to RNA processing and mRNA surveillance for putative ABCE1-interacting proteins. In addition, we identified one protein, which is directly involved in PTGS, namely translin. Together with its binding partner TRAX, translin has been purified as part of C3PO from *Drosophila* and human cells. C3PO is a Mg^2+^-dependent RNA-specific endonuclease complex that activates RISC by degrading the Ago2-nicked passenger strand of the siRNA duplex. It has been shown that translin and C3PO can bind ss-DNA and ss-siRNA, but barely interact with ds-siRNA [27,59].

Interestingly, although nuclear localization has not been reported for mammalian ABCE1, we were able to identify several nuclear proteins with co-IP experiments. This finding may indicate that similarly to yeast Rli1, the majority of ABCE1 might reside in the cytoplasm, while a minor fraction is able to enter the nucleus [17–19].

In summary, our results show that human ABCE1 is able to act as a negative regulator of RNAi in the plant *N*. *benthamiana*, in mammalian HEK293 cells and in the worm *C*. *elegans*. Therefore we have identified the first human endogenous RNA silencing suppressor. Significant reduction in 24 nt *GFP* siRNA levels in *N*. *benthamiana* and co-immunoprecipitated potential binding partners in HEK293 cells indicate that ABCE1 might function not only in PTGS but also in TGS. It will be important to determine whether mammalian ABCE1 localizes to the nucleus and its potential role in nuclear RNAi pathways. In addition, ABCE1-translin interaction and its functional significance needs further studies.

## Supporting Information

S1 Fig GFP fluorescence is enhanced in the presence of ABCE1 and AtRLI2. (PDF)

S2 Fig Expression of V5-tagged ABCE1 and P19 in HEK293 cells. (PDF)

S3 Fig ABCE1 has no significant effect on reporter gene translation. (PDF)

S1 Table Putative ABCE1-interacting proteins. HEK293 cells transfected with pABCE1-V5 and mock-transfected cells were subjected to co-IP with anti-V5 antibody. Three different sample preparation methods were used—in-gel digestion (experiment was carried out twice), in-solution digestion and FASP. The protein content of the obtained immune complexes was analyzed using LC-MS/MS. For candidate ABCE1-interacting proteins we chose those present in at least two experimental samples with experimental and control sample intensity ratio cut-off set at > 2.
(XLSX)

S2 Table Candidate ABCE1-interacting proteins that might support its function as an endogenous suppressor of RNAi. (XLSX)

S1 Text Supporting Materials and Methods. (DOCX)
