# Supplementary material for: ABCE1 Is a Highly Conserved RNA Silencing Suppressor
Source: PLoS One. 2015 Feb 6;10(2):e0116702. doi: 10.1371/journal.pone.0116702 (PMC4319951; doi:10.1371/journal.pone.0116702)
Supplement: S1 Fig — (PDF) [file pone.0116702.s001.pdf]

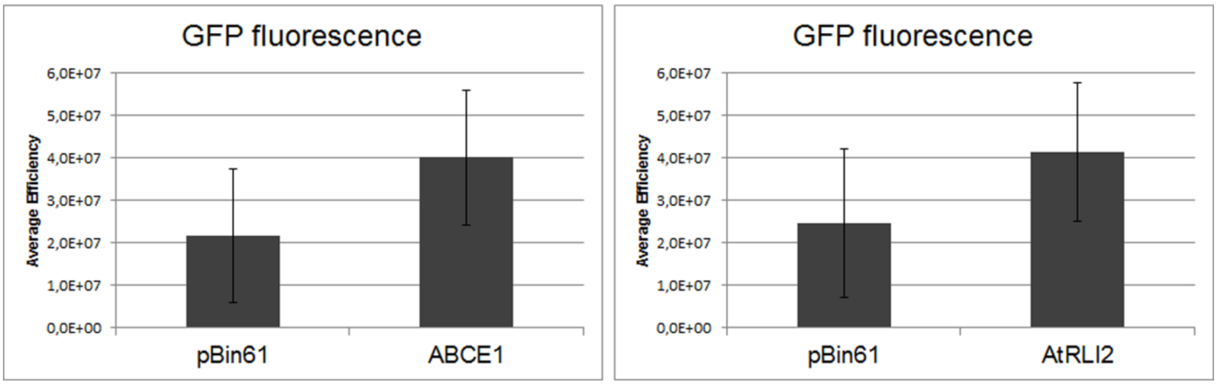

Supporting Figure S1. GFP fluorescence is enhanced in the presence of ABCE1 and AtRLI2. *Agrobacterium tumefaciens* harboring pBin61-GFP was co-infiltrated together with *A. tumefaciens* containing pBin61-ABCE1 or pBin61-AtRLI2 (indicated as ABCE1 and AtRLI2, respectively) into one half of a *Nicotiana benthamiana* (16c line) leaf blade. The other half of the leaf blade was infiltrated with the pBin61-GFP/pBin61 mixture (indicated as pBin61) for comparison. 20 leaves infiltrated with either ABCE1 or AtRLI2 were analyzed by an *in vivo* imaging system at 5 dpi. The average efficiency of GFP fluorescence was measured in the infiltrated patches and compared for each leaf separately after normalizing the values to the background fluorescence. GFP expression was higher in the presence of ABCE1 or AtRLI2 as compared to pBin61. Paired two-tail tests revealed extremely significant differences in both cases ($p < 0.0001$). Error bars correspond to standard deviations.
