# Supplementary material for: ABCE1 Is a Highly Conserved RNA Silencing Suppressor
Source: PLoS One. 2015 Feb 6;10(2):e0116702. doi: 10.1371/journal.pone.0116702 (PMC4319951; doi:10.1371/journal.pone.0116702)
Supplement: S2 Fig — (PDF) [file pone.0116702.s002.pdf]

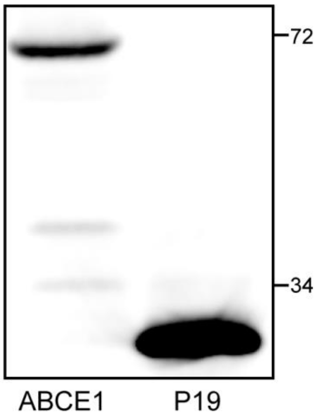

Supporting Figure S2. Expression of V5-tagged ABCE1 and P19 in HEK293 cells. FLAG-tagged ULK3 was expressed in HEK293 cells in combination with siRNA(ULK3) and plasmids encoding V5-tagged ABCE1 or P19 proteins. 30 h post-transfection cell lysates were subjected to western blotting with anti-V5 antibody. Molecular masses (in kDa) are shown on the right.
