# Supplementary material for: ABCE1 Is a Highly Conserved RNA Silencing Suppressor
Source: PLoS One. 2015 Feb 6;10(2):e0116702. doi: 10.1371/journal.pone.0116702 (PMC4319951; doi:10.1371/journal.pone.0116702)
Supplement: S3 Fig — (PDF) [file pone.0116702.s003.pdf]

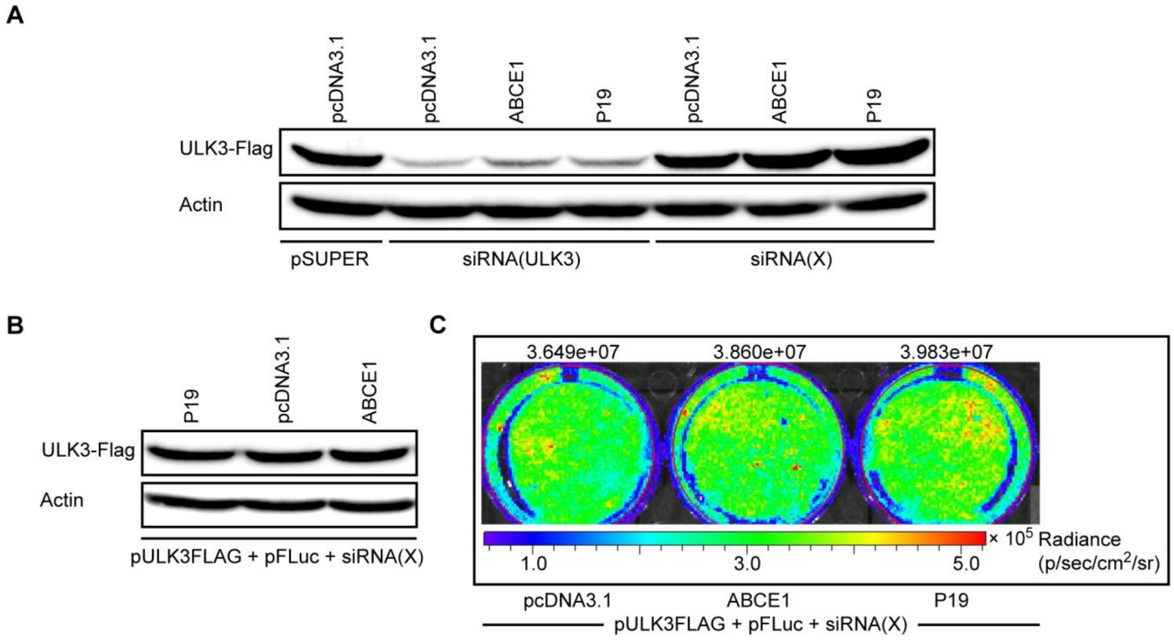

Supporting Figure S3. ABCE1 has no significant effect on reporter gene translation. (A) FLAG-tagged ULK3 was expressed in HEK293 cells in combination with empty vectors pcDNA3.1 and pSUPER or with siRNA(ULK3) or scrambled siRNA(X) and pcDNA3.1 or plasmids encoding either ABCE1 or P19 proteins. ULK3-FLAG and actin (loading control) were detected by western blotting. In silenced cells, ABCE1 was able to increase ULK3 expression levels similarly to P19, whereas in siRNA(X) transfected (non-silenced) cells neither ABCE1 nor P19 had any remarkable effect on ULK3 protein level. **(B)** ULK3-FLAG was expressed in combination with Firefly luciferase (reporter plasmid pFLuc), scrambled siRNA(X) and pcDNA3.1 or plasmids encoding either ABCE1 or P19. FLAG-tagged ULK3 and actin were detected by western blotting. ABCE1 and P19 did not have any significant effect on ULK3 expression level. **(C)** Cells were transfected as described previously and luminescence was measured in total flux (p/s). No remarkable changes in luciferase activity were observed.
