# Supplementary material for: ABCE1 Is a Highly Conserved RNA Silencing Suppressor
Source: PLoS One. 2015 Feb 6;10(2):e0116702. doi: 10.1371/journal.pone.0116702 (PMC4319951; doi:10.1371/journal.pone.0116702)
Supplement: S1 Text — (DOCX) [file pone.0116702.s006.docx]

**Supporting Materials and Methods**

**Quantification of GFP expression by *In vivo* Imaging System (IVIS)**

Different *Agrobacteria* mixtures were infiltrated into the two halves of 16c *N. benthamiana* leaf blades. At 5 dpi GFP fluorescence was quantified and analyzed by IVIS Lumina II and Living Image software (version 4.1, Perkin Elmer) as described in [[1](#_ENREF_1)] with some modifications. The GFP filter set used included a background filter (410-440 nm), an excitation filter (445-490 nm) and a GFP emission filter (515-575 nm). In every leaf the representative area of the infiltrated patches was selected, avoiding damaged tissue (e.g. due to infiltration procedure) and veins, and marked separately as the “region of interest” (ROI). In addition, ROI from non-infiltrated tissue was marked and used as a reference value for the infiltrated patches from the same leaf. The average efficiency of GFP fluorescence in ROIs represents the ratio of emitted to incident light. The values of ROIs from infiltrated patches were normalized to the background and were statistically analyzed using GraphPad InStat (version 3) software. Gaussian distribution of the values was confirmed by the Kolmogorov–Smirnov test and the p-values at 95% confidence interval were calculated using a two-tailed paired t-test.

**Transfection and luminescence measurement**

HEK293 cells were co-transfected with expression constructs pULK3FLAG encoding FLAG-tagged ULK3, pFLuc (kindly provided by I. Pata) expressing Firefly luciferase under the EF1α promoter, siRNA(X) encoding scrambled siRNAs and pcDNA3.1, pABCE1-V5 or pP19-V5 using polyethylenimine (PEI) (Inbio). Constructs pULK3FLAG, pFLuc, siRNA(X) and pcDNA3.1/pABCE1-V5/ pP19-V5 were used at the following ratio: 0.05:0.05:1:1.

D-luciferin potassium salt (Regis Technologies) was added to transfected cells (15 µg/ml final concentration) and luminescence was measured in total flux (p/s) with Xenogen IVIS Lumina II (Perkin Elmer). Subsequently the cells were lysed and subjected to western blot analysis with rabbit polyclonal anti-FLAG (1:1000, Sigma-Aldrich, catalog #F7425) and rabbit polyclonal anti-actin (1:1000, Santa Cruz Biotechnology, catalog #sc-7210) antibodies.

1. Stephan D, Slabber C, George G, Ninov V, Francis KP, et al. (2011) Visualization of plant viral suppressor silencing activity in intact leaf lamina by quantitative fluorescent imaging. Plant Methods 7: 25.
